# Supplementary material for: Genome and Pathogenicity Analysis of an NADC30-like PRRSV Strain in China’s Xinjiang Province
Source: Viruses. 2025 Mar 6;17(3):379. doi: 10.3390/v17030379 (PMC11945328; doi:10.3390/v17030379)
Supplement: Supplementary file 1 [file viruses-17-00379-s001.zip › viruses-3443299-Supplementary Table S1.pdf]

viruses

MDPI

Supplementary Table S1 Information on the referenced PRRSV strains.

No.	Isolate	GenBank Number	Country(region)	Year	No.	Isolate	GenBank Number	Country(region)	Year
1	Lelystad virus	M96262.2	Netherlands	1991	14	HENXX-1	KU950372.1	China	2014
2	ATCC VR-2332	U87392.2	USA	1992	15	IA/2014/NADC34	MF326985.1	USA	2014
3	CH-1a	AY032626.1	China	1996	16	FJZ03	KP860909.1	Fujian, China	2015
4	BJ-4	AF331831.1	Beijing, China	1996	17	HBFL-1604	MH651739.1	Hebei, China	2016
5	HuN4	EF635006.1	China	2006	18	SD53-1603	MH651744.1	Shandong, China	2016
6	TJ	EU860248.1	Tianjin, China	2006	19	NADC30	MH500776.1	China	2017
7	JXA1	EF112445.1	Jiangxi, China	2007	20	LNWK96	MG860516.1	China	2017
8	CH-1R	EU807840.1	China	2007	21	QHD1	MG687491.1	China	2017
9	NADC30	JN654459.1	USA	2008	22	GDxn1808	MT394495.1	Guangdong, China	2018
10	QYYZ	JQ308798.1	China	2011	23	RFLP 1-4-4 L 1C	MW887655.1	USA	2020
11	HENAN-HEB	KJ143621.1	China	2012	24	XJ-Z5	PQ835040	Xinjiang, China	2022
12	XW001	KF632717.1	USA	2012	25	GSFEI2-2023	PP409066.1	Gansu, China	2023
13	HENAN-XINX	KF611905.1	Henan, China	2013					
